# Maternal and neonatal outcomes of second-generation mothers in Europe: a systematic review

**DOI:** 10.1186/s12992-025-01163-y

**Published:** 2025-11-24

**Authors:** Sara Cavagnis, Davide Tarditi, Isabella Rosato, Cristina Canova

**Affiliations:** 1 Department of Medical and Surgical Sciences, University of Bologna, Bologna, Italy; 2 Unit of Biostatistics, Epidemiology and Public Health (UBEP), Department of Cardiac, Thoracic, Vascular Sciences and Public Health, University of Padua, Padua, Italy

**Keywords:** Maternal health, Migrant health, Second generation, Europe

## Abstract

**Background:**

Second-generation mothers (SGMs), born in European countries to foreign-born parents, represent a growing population. While disparities in maternal and neonatal health outcomes among first-generation migrants and natives are well documented, less is known about these outcomes among SGMs. This systematic review and meta-analysis aimed to synthesize evidence on maternal and neonatal health outcomes among SGMs, comparing them to both native-born women and first-generation migrants.

**Methods:**

We searched MEDLINE, Embase, and Scopus databases up to December 2024, for studies reporting on maternal and/or neonatal outcomes in SGMs in Europe. Eligible studies were critically appraised, and random-effects meta-analyses were conducted where possible to obtain pooled, unadjusted odds ratios.

**Results:**

A total of 19 studies were included, mostly conducted in Germany and Nordic countries. SGMs had a lower risk of C-section compared to natives (pooled OR = 0.68, 95% CI: 0.60–0.78). They also seemed to have a higher risk of late access to antenatal care (ANC) and of gestational diabetes, although not significant for the latter. For other outcomes, such as near-miss, low birth weight and preterm birth, included studies reported conflicting results or the pooled estimates were not significant. The generalizability of findings is strongly affected by the limited number of studies, data heterogeneity and underrepresentation of key migrant groups and countries.

**Conclusions:**

Structural factors, acculturation, and persistent inequalities may shape health trajectories across generations. Late access to ANC highlights that SGMs still face barriers in accessing care, despite being born and educated in host countries. Improved data collection, disaggregation by parental background, and attention to social determinants are essential to better understand and address the needs of this growing population.

**Supplementary Information:**

The online version contains supplementary material available at 10.1186/s12992-025-01163-y.

## Background

In recent decades, migration dynamics have reshaped the demographic structure of European Union (EU) countries, leading to a growing number of individuals with a migration background, including second-generation people. Following the European Commission’s definition, second-generation individuals are those who were born in and reside in a country to which at least one parent previously migrated [1]. In 2023, 14.5% of people aged 15 to 74 years and residing in the EU were born abroad, and 7.3% were children of parents born abroad (i.e., second-generation migrants) [2]. Notably, migration has a significant impact on birth patterns: in 2023, 23% of all newborns in the EU had mothers born abroad—a rise of 5% points compared to 2013. This translates to approximately 840,000 births annually. Among these mothers, 74.2% came from non-EU countries [3].

A migrant background is widely recognized as a social determinant contributing to health disparities, due to various factors, including language and cultural barriers, legal status, stigma and segregation, and, for women, gender-related power imbalances [4, 5]. In the field of maternal and child health, several reviews report that migrant women have less access to care during pregnancy, experience a higher number of complications during childbirth, and are at greater risk of maternal mortality [6–8]. Furthermore, migrant mothers’ children have a greater risk of preterm birth, low birth weight (LBW), and mortality within the first 30 days of life [7, 9].

Inequalities and differences in maternal and neonatal health outcomes among second-generation mothers (SGMs) and their children have been less extensively explored, possibly due to difficulties in defining this population and the relatively recent immigration history in some EU countries. One systematic review on birth weight differences among children of first-generation and second-generation migrant mothers highlighted that children born to SGMs had an increased risk of LBW (OR 1.21, 95% CI: 1.15–1.27) [10]. However, this review only included studies from the USA and the United Kingdom (UK); these countries have a long history of immigration, and migrant populations whose countries of origin differ from those of migrants in other European countries. Information on the outcomes of SGMs and their children in Europe is not consolidated, and to the best of our knowledge, no other systematic reviews on this topic are available. Characterizing the health of second-generation women during pregnancy and childbirth is crucial to guide evidence-based policy decisions and reduce persistent health inequalities in increasingly diverse European societies.

The aim of this study was to systematically review the existing published literature on the use of healthcare services and health outcomes during pregnancy, childbirth, and the neonatal period among SGMs residing in EU, in the European Free Trade Association (EFTA) countries, and in the UK.

## Methods

### Search strategy and inclusion criteria

We conducted a systematic review following the guidelines outlined by the Preferred Reporting Items for Systematic Reviews and Meta-Analyses (PRISMA) statement [11]. The review protocol was registered on PROSPERO (CRD42025633284).

The population of interest comprised SGMs and their newborn children living in the European Union, in the EFTA countries, and in the UK. A complete list of the included countries is provided in Table S1, Additional File 1. The comparison group included either native or first-generation migrant mothers and their newborn children. We included studies investigating maternal healthcare access and use (e.g., delayed first visit), maternal health outcomes (e.g., mode of delivery, gestational diabetes) and newborn health outcomes (e.g., LBW, Apgar score). The list of outcomes included in the search was based on the available literature on first-generation migrants and the researchers’ clinical experience; to broaden the scope of the review, we aimed to include a wide range of outcomes related to maternal and neonatal health. A complete list of outcomes included in the search and the Population-Exposure-Comparison-Outcome (PECO) framework is available in Table S2, Additional File 1.

On December 10, 2024, relevant documents were identified by searching the electronic databases PubMed, Scopus, and Embase, using a comprehensive search strategy. The search string included terms related to second-generation migrants, reproductive and maternal health outcomes and European countries with spelling variations, to ensure the capture of all relevant studies. Keywords referring to the population and outcomes of interest were searched in the title and abstract, while terms related to the setting were searched as free-text across all fields. The detailed search strategy for each included database is reported in Table S3, Additional File 1.

We included peer-reviewed, quantitative observational studies published in English or Italian and conducted in the European Union (EU) or the EFTA. We selected only papers published in or after the year 2000. This timeframe was chosen to reflect the most recent trends in maternal and neonatal health outcomes among second-generation women in Europe, as well as changes in migration patterns, healthcare policies, and public health strategies. We excluded qualitative papers, conference abstracts, and reviews. Studies that only reported ethnicity or those that grouped first-generation migrants and SGMs were excluded.

The screening was conducted using Covidence software [12]. After removing duplicates, two independent reviewers (S.C and D.T.) evaluated the articles by title and abstract against the inclusion and exclusion criteria. Any discrepancies were discussed and resolved, with the intervention of a third reviewer (I.R.) when necessary. Then, the same two reviewers independently examined the full texts of potentially eligible studies, resolving disagreements through discussion with a third reviewer. The reasons for exclusion were recorded. In addition, the reference lists of included studies were hand-searched to identify further relevant publications, which were included when eligible.

### Data extraction and analysis

For each study, we extracted detailed information on the following:

- Study characteristics: title, first author, publication year, journal, study design, data sources, language used for data collection, period of data collection, country, and study setting;
- Population characteristics: sample size and group size, mean or median age, country or geographical area(s) of parental origin, definition of second generation, proportion of SGMs with low education (as defined in the primary study), occupational status, partnering status, parity;
- Outcomes: definition, measure of the association, statistical model used, confounding factors considered, subgroup analyses, number of cases overall and in each subgroup.

For each outcome considered in the review and reported in at least three independent studies, we conducted a meta-analysis to estimate pooled unadjusted odds ratios (ORs) and corresponding 95% confidence intervals for the association between second-generation status (compared with native-born women) and maternal or neonatal outcomes. When unadjusted ORs were not directly reported, we extracted raw data (e.g., counts of events and total sample sizes) from main tables and supplementary materials to recalculate them. We prioritized unadjusted ORs to ensure comparability across studies, as adjustment variables often differed widely and were not consistently reported; also, the aim of the study was to examine the differential risk between second-generation and native-born women and not causal mechanisms.

Given the expected methodological and contextual heterogeneity across studies—including differences in populations, healthcare systems, and outcome definitions—we applied a random-effects model for all meta-analyses. Statistical heterogeneity was assessed using the I^2^ statistic, but we did not use it as a criterion to select between fixed- and random-effects models, as low I^2^ values do not necessarily imply clinical or methodological homogeneity. Where possible, we also calculated unadjusted ORs for the comparison between second-generation and first-generation migrant women as a secondary analysis. When more than one paper was based on the same data source, we described all published studies but included only the study with the largest sample size for each outcome in the synthesis. A narrative synthesis was used for outcomes reported in fewer than three studies. The risk of bias was assessed by two independent reviewers using the Johanna Briggs Institute Critical Appraisal Tools for nonrandomized studies [13]. Instead of assigning an overall quality score to each study, we provided a domain-specific assessment of each item, allowing us to identify domains of methodological strength and potential bias.

## Results

**Fig. 1:**
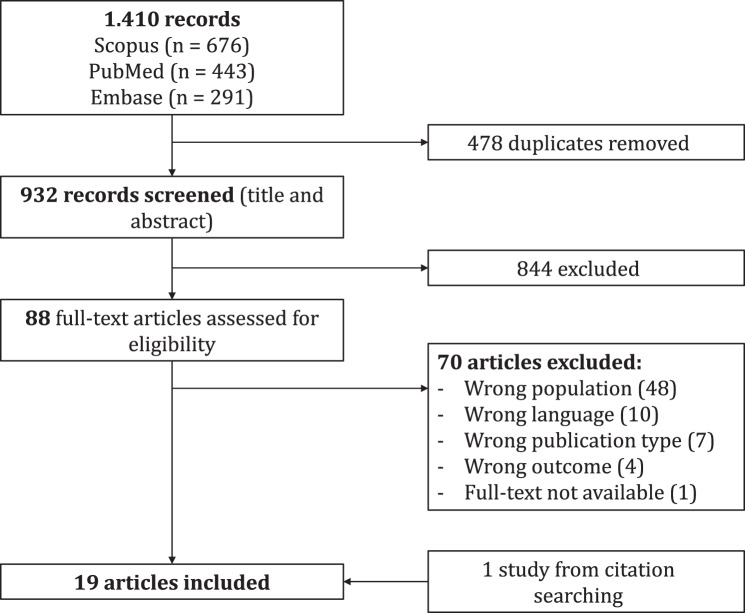
Prisma flow diagram

A total of 1,410 studies were imported for screening in Covidence, of which, after deduplication, 932 were screened by title and abstract (Fig. 1). 88 full-text studies were assessed for eligibility, of which 18 were included in the review. The reasons for exclusion after the full-text review phase are listed in Fig. 1. An additional study was identified through citation searching in the included studies.

### Study characteristics

Table 1 presents the characteristics of the included studies. Of the 19 studies included, 11 were conducted in Germany, 3 in Sweden, 2 in Norway, 2 in the Netherlands and 1 in France [14–32]. Notably, the 19 papers were based on 11 data sources, because 10 reported on the same two populations 8 based on a study conducted in Germany in 2011–12 and two on the same Swedish cohort. 11 studies were cross-sectional, while 8 were cohort studies. 5 studies considered the whole country’s population; the rest were conducted in maternity hospitals. The majority of the studies (*n* = 12) were conducted after 2010, but 4 cohort registry-based studies used data beginning in 1989 [29], 1995 [15] and 1998 [30, 32].

**Table 1:** Characteristics of the included studies

Study	Study design and data collection	Period of data collection	Country, context	Sample size (N and % of total for SGMs)	Definition of second generation	Countries or areas of origin of SGMs	Age (natives; SGMs)	Low education (natives vs SGMs)
Choté A., 2014 [14]	Cross-sectional questionnaire	2002–2004	Netherlands, midwife practices	845 (219, 25.9%)	At least one parent born abroad	Turkish (42%), Cape Verdean (21%), Moroccan (12%), Surinamese Creole (11%), Surinamese Hindustani (9%) and Dutch Antillean (6%)	Mean age: NA; 23.9	NA vs 12.3%
Sørbye IK, 2014 [15]	Cohort, registry-based	1995–2010	Norway, whole country	723,045 (1,801, 0.2%)	Both parents born abroad	Pakistan	Mean age: 29.3; 25.5	15.8% vs 37.3%
David M., 2014* [16]	Cross-sectional questionnaire linked with hospital database	2011–2012	Germany, three secondary and tertiary-care maternity hospitals in Berlin	4,598 (580, 12.6%)	Both parents born abroad	Turkey	Mean age: 30.9; 28.6	3% vs 7.1%
David M., 2015* [17]	Cross-sectional questionnaire linked with hospital database	2011–2012	Germany, three secondary and tertiary-care maternity hospitals in Berlin	7,100 (958, 13.5%)	Both parents born abroad	NA (from same datasource: Turkey 60%, Lebanon 16%, others)	Mean age: 30.8; 27.6	3.3% vs 9.3%
Boerleider A., 2015 [18]	Cohort, questionnaire and registry-based	2009–2011	Netherlands, midwife practices	3,300 (99, 3.0%)	At least one parent born abroad	NA	85%; 94% in category 20-35y	14.3% vs 20.4%
Reiss K., 2016* [19]	Cross-sectional questionnaire linked with hospital database	2011–2012	Germany, three secondary and tertiary-care maternity hospitals in Berlin	4,859 (697, 14.3%)	Both parents born abroad	NA (from same data source: Turkey 60%, Lebanon 16%, others)	Mean age: 30.6; 27.6	3.2% vs 8.4%
Razum O, 2017* [20]	Cross-sectional questionnaire linked with hospital database	2011–2012	Germany, three secondary and tertiary-care maternity hospitals in Berlin	6,391 (839, 13.1%)	Both parents born abroad	Largest group: Turkish	Mean age: 30.8; 27.6	3.2% vs 9.2%
Bakken K, 2017 [21]	Cohort, registry-based	2006–2013	Norway, low-risk maternity ward	8,524 (86, 1.0%)	Unclear	Pakistan	Mean age: 31.7; 27.6	27.4% vs 69.7%
David M., 2017* [22]	Cross-sectional questionnaire linked with hospital database	2011–2012	Germany, three secondary and tertiary-care maternity hospitals in Berlin	6,388 (928, 14.5%)	Both parents born abroad	Turkey (60%), Lebanon (16%), others	Median age: 31; 27	3% vs 9.4%
El-Khoury Lesueur F., 2018 [23]	Cohort, interviews and medical records	2011	France, whole country	16,473 (2,179, 13.2%)	At least one parent born abroad	53% EU/France, 32% North Africa and Turkey, 8% Sub-Saharan Africa, 4% Eastern Europe and Asia, 3% other/missing	NA	NA
David M., 2018* [24]	Cross-sectional questionnaire linked with hospital database	2011–2012	Germany, three secondary and tertiary-care maternity hospitals in Berlin	3,765 (946, 25.1%)	At least one parent born abroad	not available (from same data source: Turkey 60%, Lebanon 16%, others)	NA; 65% in category 18-29y	NA vs 38.2%
David M., 2019* [25]	Cross-sectional questionnaire linked with hospital database	2011–2012	Germany, three secondary and tertiary-care maternity hospitals in Berlin	6,767 (889, 13.1%)	Both parents born abroad	NA (from same data source: Turkey 60%, Lebanon 16%, others)	41%; 66% in category 18-29y	15% vs 38%
Breckenkamp J., 2019* [26]	Cross-sectional questionnaire linked with hospital database	2011–2012	Germany, three secondary and tertiary-care maternity hospitals in Berlin	4,497 (655, 14.6%)	Both parents born abroad	Turkey (61%), Lebanon (16%), others	Mean age: 30.9; 27.6	2.8% vs 8.9%
Seidel V., 2020 [27]	Cross-sectional questionnaire	2020	Germany, one maternity ward in Berlin	460 (62, 13.5%)	At least one parent born abroad	NA	Median age: 33; 29	2.2% vs 3.2%
Miani C., 2020 [28]	Cohort, questionnaire linked with hospital database	2013–2016	Germany, three maternity hospitals	881 (92, 10.4%)	Both parents born abroad	NA	NA	NA
Aradhya S., 2022 [29]	Cohort, registry-based	1989–2012	Sweden, whole country	246,642 (21,971, 8.9%)	Other (mother born abroad)	Other Nordic countries (58%), Eastern Europe (15%), non-Western countries (15%), Western countries (12%)	56%; 60% in category 20–29	50% vs 59%
Wändell P, 2023^ [30]	Cohort, registry-based	1998–2018	Sweden, whole country	989,986 (171,210, 17.3%)	At least one parent born abroad	Other nordic countries (42%), other Europe (30%), Africa (3%), America (5%), Asia (19%), other (1%)	44%; 49% in category 20-29y	20.8% vs 31.2%
Lee M., 2023 [31]	Cross-sectional questionnaire linked with hospital database	2018–2020	Germany, one maternity ward in Berlin	896 (160, 17.9%)	At least one parent born abroad	NA	Mean age: 33.3; 30.5	11.5% vs 31.3%
Wändell P, 2024^ [32]	Cohort, registry-based	1998–2018	Sweden, whole country	1,255,332 (171,210, 13.6%)	At least one parent born abroad	Other Nordic countries (42%), other Europe (30%), Africa (3%), America (5%), Asia (19%), other (1%)	44%; 49% in category 20-29y	20.8% vs 31.2%

Notes: SGMs: second-generation mothers; NA: not available; y: years

* Publications from the same study and data sources

^ Publications from the same study and data sources

The exposure of interest (being a second-generation migrant) was defined according to responses to questionnaires in 14 studies, and by using information retrieved from registries in 5 studies. The criteria used to identify SGMs varied: nine studies defined as second generation those with both parents born abroad, 8 studies included those with at least one parent born abroad, and 1 study only included those whose mother was born abroad, irrespective of the father’s origin. Four studies included third-generation mothers within the second-generation group (i.e., the grand-daughters of migrants) [16, 17, 20, 28].

Participants numbers ranged from 460 to 1,255,332 (with a median of 6,388 participants); SGMs comprised between 0.2% and 25.9% of the study population. In all the included studies, SGMs, compared with native-born mothers, were younger and more frequently had a lower level of education. In some studies, SGMs were selected according to their area of origin: Turkey [16] and Pakistan [14, 15].

Most studies reported on multiple outcomes, including maternal healthcare access and use (late antenatal care (ANC), number of ANC visits, use of analgesia during labour), maternal health outcomes (gestational diabetes, maternal hypertension, pre-eclampsia, induction of labour, C-section, near-miss events as defined in Table S2, Additional file 1), and newborn outcomes (LBW, mean birth weight, macrosomia, Apgar score, access to neonatal intensive care unit (NICU), small for gestational age (SGA), stillbirth, preterm birth). Some outcomes that were included in the search strategy were not reported in any study: antenatal screening, vaccination during pregnancy, maternal ICU admission, maternal mortality, neonatal mortality and congenital malformations. A detailed list of the outcomes reported in each included study can be found in Table S4, Additional File 1.

### Risk of bias assessment

The risk of bias assessment for each study is presented in Tables S5 and S6, Additional File 1.

All cross-sectional studies adequately defined the inclusion criteria and described the included subjects and the setting. All studies employed valid methodologies for measuring exposure and outcomes and stated their strategy to address confounding factors. In two studies, there was a risk of selection bias [14, 27], due to low response rate and lack of informed consent.

The cohort studies were found to be of overall high quality, with appropriate exposure and outcome definitions and statistical analysis. Three studies had a moderate risk of selection bias due to a low response rate, the language of the data collection tools and the recruitment strategy [18, 23, 28]; two studies had a small sample size [18, 21] one of which did not adjust for all relevant covariates [21].

### Maternal and neonatal health outcomes: meta-analyses results

The results from the meta-analyses, conducted including only maternal and neonatal health outcomes reported in at least three studies, are presented in Table 2, which includes comparisons between SGMs and native mothers, as well as between SGMs and first-generation mothers, where reported.

**Table 2:** Results of meta-analyses by outcome, comparing SGMs with both natives and migrants, when 3 or more studies were included

Section	Outcome	Countries of included studies (references)	Comparison group	Pooled OR (95% CI)	**Heterogeneity (I**^**2**^)
Healthcare access	Use of analgesia during labour	Germany, Norway [20, 21, 31]	Natives	1.07 (0.58–1.97)	92%
			1st generation	1.44 (1.14–1.82)	24%
Maternal health	Gestational diabetes	France, Germany, Norway, Sweden [21, 23, 25, 32]	Natives	1.94 (0.64–5.88)	92%
			1st generation	0.79 (0.57–1.10)	73%
	Induction of labour	Germany, Norway [19, 21, 31]	Natives	1.04 (0.88–1.23)	0%
			1st generation	1.06 (0.81–1.38)	9%
	C-section (overall)	Germany, Norway [17, 21, 28, 31]	Natives	0.68 (0.60–0.78)	0%
			1st generation	0.90 (0.73–1.10)	0%
	Emergency c-section	Germany, Norway [17, 21, 28, 31]	Natives	0.77 (0.65–0.92)	0%
			1st generation	1.06 (0.90–1.25)	10%
	Elective c-section	Germany, Norway [17, 21, 31]	Natives	0.69 (0.56–0.85)	0%
			1st generation	0.55 (0.25–1.23)	77%
Neonatal health	Low birth weight	Germany, Norway, Sweden [21, 22, 29]	Natives	1.37 (0.44–4.29)	89%
	Preterm birth	Germany, Norway [21, 24, 31]	Natives	1.80 (0.61–5.33)	93%
			1st generation	1.42 (0.88–2.29)	54%
	Access to NICU	Germany, Norway [17, 21, 31]	Natives	0.96 (0.47–1.99)	84%
			1st generation	0.90 (0.43–1.88)	70%

When examining healthcare access and use, we identified three studies that assessed the use of analgesia during labour [20, 21, 31]. SGMs did not differ from natives (pooled OR 1.07, 95% CI: 0.58–1.97), while they were more likely to use analgesia than first-generation mothers (pooled OR 1.44, 95% CI: 1.14–1.82).

In the domain of maternal health, we explored multiple outcomes. Six studies examined gestational diabetes, from four different data sources [21, 23, 25, 26, 30, 32]. Overall, four studies found that SGMs had a higher risk of gestational diabetes compared to natives (pooled OR: 1.94, 95%CI: 0.64–5.88), and a lower risk compared to first-generation migrants (pooled OR: 0.79, 95% CI: 0.57–1.10), although both results were not significant (Table 2). One large registry-based cohort study included in the meta-analysis specifically focused on gestational diabetes risk among SGMs [32]: this study revealed an increased risk of gestational diabetes (hazard ratio (HR): 1.27; 99% CI: 1.19–1.36), particularly in women whose parents originated from Eastern Europe, Central Europe, Africa, or Asia.

Three studies reported on the induction of labour with mixed findings [19, 21, 31]. The pooled OR was 1.04 (95% CI: 0.88–1.23). No significant difference resulted with first-generation migrants (pooled OR 1.06, 95% CI: 0.81–1.38) (Table 2).

Four studies from different data sources assessed C-section rates [17, 21, 28, 31]. SGMs were at lower risk for a C-section (elective, emergency, and overall) compared to natives (pooled OR for overall C-section: 0.68, 95% CI: 0.60–0.78). There were no significant differences in C-section rates between second- and first-generation (Table 2).

For neonatal outcomes, we considered preterm birth, admission to NICU, and LBW. Three studies investigated preterm birth [21, 24, 31]. The pooled OR was 1.80 (95% CI: 0.61–5.33). No significant difference was found comparing with first-generation migrants (pooled OR: 1.42, 95% CI: 0.88–2.29) (Table 2).

Three studies reported on NICU admissions [17, 21, 31], with contrasting results. The pooled OR was 0.96 (95% CI: 0.47–1.99) for second-generation children compared to natives. Similarly, there was no significant difference between first- and second-generation newborns regarding NICU access (Table 2).

Three studies reported on LBW (birth weight < 2500 g), with contrasting results [21, 22, 29]. The pooled OR was 1.37 (95% CI: 0.44–4.29) (Table 2). There was an insufficient number of studies to compare first-generation and SGMs.

### Healthcare access and maternal and neonatal outcomes not included in the meta-analysis

Additional outcomes were not incorporated into the meta-analyses, either because they were reported in fewer than three studies or due to considerable heterogeneity in outcome definitions across available studies.

With respect to the healthcare access and use domain, five studies examined late ANC access, including two from the same population [14, 18, 22, 24, 27], comparing SGMs to both natives and first-generation migrants. Two studies found that SGMs accessed care later than natives [18, 27]. Additionally, late access in SGMs was reported to be less frequent compared to first-generation migrants in four studies [14, 18, 24, 27]. David M and colleagues reported on the mean gestational age (GA) at the first antenatal check-up, which was 9 weeks for both natives and SGMs and 9–10 weeks (depending on the area of origin) for first-generation migrants [22].

In terms of the mean number of antenatal visits, five studies were reviewed [16, 18, 25, 27, 31], including two with overlapping populations. Descendants of immigrants had an insufficient number of ANC visits more frequently than natives in four studies [18, 25, 27, 31]; one German study on those of Turkish origin found no significant difference [16]. The number of visits was consistently higher for SGMs than migrants in all studies, except in the study by Boerleider et al. [18].

With respect to additional maternal outcomes, two studies from the same Swedish cohort investigated hypertension during pregnancy [30, 32]. SGMs had a lower risk than natives, with a fully-adjusted HR of 0.88 (99% CI: 0.86–0.89); a reduced risk was also observed when stratifying by type of hypertension (gestational hypertension, pre-eclampsia and eclampsia). The lower risk observed in second-generation women was not as pronounced as that of first-generation women, who, compared with natives, had a fully-adjusted HR of 0.69 (95% CI: 0.66–0.73).

Only one German study investigated near-miss events, which included HELLP syndrome, eclampsia, occurrence or threat of uterine rupture, postpartum haemorrhage > 1000 ml, sepsis, peri-partal hysterectomy in connection with a C-section, cardiovascular complications, and lung embolism [25]. While the proportion of near-miss events was slightly higher in SGMs (2.2%) compared to natives (1.9%), the difference was not significant after adjusting for relevant covariates, with an adjusted OR of 1.3 (95% CI: 0.76–2.21).

With respect to additional neonatal outcomes, four studies examined mean birth weight, showing results consistent with the LBW findings [21, 22, 29, 31]. Children of second-generation Pakistani mothers weighed on average 292 g less than those of native Norwegian mothers [21]. Aradhya et al also stratified the results by area of origin: SGMs from non-Western countries had children whose weight was significantly lower than natives (−147 g mean difference, 95% CI − 163 g to − 132 g) [29]. Two studies found no significant differences in mean birth weight [22, 31].

Being small for gestational age (SGA) was addressed in one study [22], which found a slightly lower incidence among SGMs compared to natives (7.9% vs 8.4%), although the association was not statistically significant (adjusted OR: 0.81, 95% CI: 0.61–1.08).

One German study reported on foetal macrosomia (birthweight ≥ 4.0 kg) and found a lower prevalence among SGMs (9.0%) compared to natives (11.9%) [26].

Two German studies evaluated low Apgar score at 5 minutes [22, 31]. Both showed a lower proportion of low Apgar scores for the children of SGMs compared to the children of natives (0% vs 1.3% and 1.6% vs 2.3%, respectively).

One study on second-generation Pakistani mothers in Norway highlighted an increased risk of stillbirth, with an adjusted RR of 2.19 (95% CI: 1.13–4.24) [15].

## Discussion

This systematic review explored maternal and neonatal health outcomes among SGMs in Europe. A total of 19 studies met our inclusion criteria; however, 10 of these relied on two datasets from Germany and Sweden, resulting in a total of 11 unique study populations. Only a limited number of countries were represented in the included studies, and key European countries, with both short and long-standing migration histories (e.g., Italy, Spain, Greece and the UK), were absent. There was substantial heterogeneity in the parental countries of origin of SGMs, with countries such as Turkey, Lebanon, Pakistan, and other European countries being well represented, while other areas, such as Sub-Saharan Africa, South America, and Eastern Asia, were underrepresented or entirely absent. Some outcomes included a-priori in our search, like maternal and neonatal mortality, were not assessed in any of the included studies. Moreover, variation in outcome definitions across studies often limited the feasibility of conducting meta-analyses.

The review shows heterogeneous findings regarding the health outcomes of SGMs. For certain outcomes, such as late ANC and gestational diabetes, SGMs appear to have a higher risk compared to native women, but a lower risk compared to first-generation migrants. Conversely, SGMs had a reduced risk of hypertension during pregnancy, lower C-section rates (for both elective and emergency C-section), and decreased risk of low Apgar scores, when compared to natives. For other outcomes, such as having a SGA newborn, near-miss events, LBW, preterm birth, and NICU admissions, results were inconclusive or showed no statistically significant difference. Subgroup analyses conducted in primary studies – particularly those focusing on Pakistani SGMs – suggested potential risks in specific ethnic or migratory groups. However, the limited number of disaggregated studies precluded definitive conclusions.

Existing evidence across Europe shows that migrants and ethnic minorities generally experience poorer ANC access [6, 33, 34]. Several factors have been associated with delayed first contact with healthcare services, including predisposing factors such as age and parity, and enabling factors such as education level, occupation, language proficiency, and self-perceived health. Thus, healthcare access among SGMs may be shaped by structural barriers or vulnerabilities that persist across generations.

Other outcomes examined in this review, such as gestational hypertension and diabetes are influenced by predisposing factors including age, genetic predisposition, and health behaviours (e.g., diet, smoking and physical activity) [35, 36]. These factors can also affect newborn weight; however, the differences reported in the included studies may also be related to physiological variations rather than adverse health outcomes [37, 38]. Acculturation might play a dual role: while facilitating healthcare access, it might introduce unhealthy behaviours, common in host populations [39]. Moreover, such behaviours might develop in response to chronic stressors, such as discrimination and economic disadvantage [40].

The observed lower C-section rates among SGMs likely reflect their younger maternal age, which is a known protective factor [41, 42]. Besides, the mothers’ wishes, which can be shaped by culture and country of origin [43], and physicians’ attitudes, might also play a role in the observed differences [44].

This study has several strengths. To the best of our knowledge, this is the first systematic review focused on the health of SGMs and their newborns in Europe. By including a wide range of outcomes, this review provides a comprehensive overview of maternal and neonatal health of this population. Nonetheless, this breadth highlighted important limitations of the existing evidence, with only eleven study populations included in the review; this severely limits generalizability to Europe as a whole. Moreover, direct comparisons across European countries may be inappropriate, given the variability in migration histories and population structures. Many outcomes were reported in only one or two studies, making it challenging to synthesize findings. When feasible, we conducted meta-analyses, which, however, should be interpreted cautiously due to variability in the definition of outcomes, setting, and country of origin of SGMs, and substantial statistical heterogeneity. Another limitation lies in the selection of studies written only in English or Italian, which might have led to the exclusion of relevant studies published in other languages. Due to inconsistent reporting of adjustment variables across studies, pooled ORs were calculated as unadjusted. This approach offers a more comparable and realistic representation of the health status of SGMs and their newborns than adjusted estimates. However, it limits causal inference, because it doesn’t allow to account for relevant confounders, such as maternal age, education, or socioeconomic status. This can, in fact, explain some of our findings, as maternal age is a well-established determinant of many maternal health outcomes, with younger women generally experiencing fewer complications than older mothers [45]. In all included studies, SGMs were younger than their native-born counterparts, possibly reflecting earlier childbearing patterns in this group. Another explanation for this age difference could lie in the relatively recent migration history in some areas of Europe, where second-generation women are still predominantly in younger reproductive age groups. In both cases, as second-generation women age and their fertility patterns evolve, the protective effect associated with younger maternal age may diminish, potentially resulting in less favourable outcomes for future generations of SGMs [46].

Research on second-generation individuals entails specific methodological challenges, the foremost being that the criteria used to define second-generation migrants are not always uniform, as demonstrated by the results of this review. Regardless of the definition used, accurately identifying the second generation requires data not only on their own country of birth, but also on that of their parents—information that is not routinely collected in many datasets. This limitation may contribute to the small number of available studies. While primary data collection through surveys helps identify this group, it can be costly and time-consuming and may result in small sample sizes. Conversely, administrative data sources are constrained by the availability of population registries that include detailed migration history information, a feature present only in certain European countries, particularly those in Northern Europe. The limited availability of disaggregated data on migration background reflects a broader lack of attention to the health of second-generation migrants. This gap is not only a methodological issue—it is indicative of a systemic neglect of a population that, while born in Europe, may continue to face disadvantage and social exclusion [47, 48]. It is essential to address these data limitations, to improve understanding and ensure that the health needs of the second generation are adequately described and addressed.

Secondly, it is possible that a greater number of studies, particularly from countries with an old migratory history, focus on ethnicity rather than migration background. Although correlated, migration experience and ethnicity are two distinct constructs and are likely to influence health through different, intertwined pathways. Second-generation individuals are not directly exposed to the migration experience; however, they might be affected by their parents’ experience, including challenges related to legal status, socioeconomic disadvantage, and cultural adaptation. These experiences can lead to intergenerational trauma, which in turn can influence behaviours and health [49]. Moreover, children of migrants often grow up navigating between two cultural contexts, which can lead to stress, mental health issues and risk behaviours, particularly in adolescence and early adulthood [50]. Some second-generation individuals also belong to ethnic minorities within their country of birth, and may experience systemic racism and discrimination, which can negatively influence both general health and maternal health outcomes [51–53]. In this review, several studies did not stratify results by parental area of origin, potentially masking important variations within the second-generation group. Aggregating second-generation individuals with diverse backgrounds—particularly those of European versus non-European origin—may obscure subgroup-specific risks, given that those with European ancestry are more likely to exhibit health outcomes similar to the native population.

Given the complex interaction of factors influencing the health of individuals with a migratory background, and the growing contribution of second generations to overall birth rates in Europe, further research is essential to better understand maternal and neonatal outcomes in this population. This is particularly relevant in light of evidence suggesting adverse health outcomes among second-generation individuals. A review reported higher mortality rates in both early life and adulthood in second-generation individuals compared to both natives and first-generation migrants, particularly when their parents were born outside of Europe [54]. Furthermore, a European survey showed that second-generation migrants had the highest prevalence of poor self-reported health compared with both migrants and natives, with disparities being especially marked among women of lower socio-economic status [55].

## Conclusions

In conclusion, the outcomes of SGMs reflect a unique interplay of demographic, social and structural factors. Future research should be conducted in other European countries, expand the scope of investigation to include other maternal outcomes, and should strive to disaggregate results by parental background (ethnicity or specific country of birth). Moreover, analyses should explicitly account for structural and contextual factors that shape health trajectories. Researchers and policymakers should prioritize the improved identification of second-generation individuals within administrative data systems. Such efforts are critical to generating robust evidence and guiding the development of equitable health policies in increasingly diverse European societies.

## Electronic supplementary material

Below is the link to the electronic supplementary material.

supplementary material 1

## Data Availability

Data sharing is not applicable to this article as no datasets were generated or analysed during the current study.

## Abbreviations

ANC: Antenatal care
EFTA: European Free Trade Association
EU: European Union
HR: Hazard ratio
LBW: Low birth weight
NICU: Neonatal intensive care unit
OR: Odds ratio
SGA: Small for gestational age
SGM: Second-generation mothers
