# Supplementary material for: Maternal and neonatal outcomes of second-generation mothers in Europe: a systematic review
Source: Global Health. 2025 Nov 24;22:2. doi: 10.1186/s12992-025-01163-y (PMC12764005; doi:10.1186/s12992-025-01163-y)
Supplement: Supplementary file 1 — supplementary material 1 [file 12992_2025_1163_MOESM1_ESM.docx]

**Supplementary materials**

**Table S1.** Included countries

| **EU countries** | Austria, Belgium, Bulgaria, Croatia, Cyprus, Czech Republic, Denmark, Estonia, Finland, France, Germany, Greece, Hungary, Ireland, Italy, Latvia, Lithuania, Luxembourg, Malta, Netherlands, Poland, Portugal, Romania, Slovakia, Slovenia, Spain, Sweden |
| --- | --- |
| **EFTA countries (extra EU)** | Iceland, Liechtenstein, Norway, Switzerland |
| **Other European countries** | UK |

**Table S2.** PECO framework

| Population | People living in European countries (EU+EFTA) |
| --- | --- |
| Exposure | Migratory background (being a second-generation mother) |
| Comparison | The native population of the residing country or first-generation migrants |
| Outcomes | - Maternal healthcare use (delayed first visit, number of antenatal visits, maternal vaccinations, antenatal screening) - Maternal health outcomes (maternal mortality, mode of delivery (c-section), labour pain management, preterm birth, gestational diabetes, pre-eclampsia, eclampsia and other hypertension disorders, maternal ICU admission, maternal near miss*) - Newborn outcomes (low birth weight, mean birth weight, small for gestational age, large for gestational age, Apgar score, congenital malformations, neonatal mortality, stillbirth, access to NICU) |

* Near miss was defined as any event in which a woman nearly dies of a complication during pregnancy and childbirth.

**Table S3.** Search strings (search date: 10/12/2024)

| **Database** | **String** |
| --- | --- |
| Pubmed | (Europe OR "European Union" OR EU OR "European Free Trade Association" OR EFTA OR Euro* OR Austria OR Belgium OR Bulgaria OR Croatia OR Cyprus OR "Czech Republic" OR Denmark OR Estonia OR Finland OR France OR Germany OR Greece OR Hungary OR Iceland OR Ireland OR Italy OR Latvia OR Liechtenstein OR Lithuania OR Luxembourg OR Malta OR Netherlands OR Norway OR Poland OR Portugal OR Romania OR Slovakia OR Slovenia OR Spain OR Sweden OR Switzerland OR "United Kingdom" OR UK OR England OR Scotland OR Wales OR "Northern Ireland") AND (((second-generation OR "second generation" OR "descendant*" OR "children" OR "offspring") AND (migrant* OR immigrant* OR "foreign-born parent*" OR refugee*)) OR "parental migration" OR "migrat* background*" OR "migrant background*" OR "family migrat* history" OR "immigrant descen*" OR "migrant descen*") AND ("pregnancy outcome*"[Title/Abstract] OR "neonatal outcome*"[Title/Abstract] OR "perinatal outcome*"[Title/Abstract] OR "maternal outcome*"[Title/Abstract] OR "maternal mortalit*"[Title/Abstract] OR "maternal mortality"[MeSH Terms] OR "maternal death*"[Title/Abstract] OR "maternal death"[MeSH Terms] OR "pregnancy-related mortalit*"[Title/Abstract] OR "pregnancy-related death*"[Title/Abstract] OR "pregnancy-associated mortalit*"[Title/Abstract] OR "maternal near-miss"[Title/Abstract] OR "severe maternal morbidit*"[Title/Abstract] OR "severe acute maternal morbidit*"[Title/Abstract] OR eclampsia*[Title/Abstract] OR "eclampsia"[MeSH Terms] OR preeclampsia*[Title/Abstract] OR "pre eclampsia*"[Title/Abstract] OR "Pregnancy toxemia*"[Title/Abstract] OR "toxemia of pregnanc*"[Title/Abstract] OR "pre-eclampsia"[MeSH Terms] OR "gestational diabete*"[Title/Abstract] OR "maternal diabete*"[Title/Abstract] OR "pregnancy diabete*"[Title/Abstract] OR "gestational hyperglycemia" [Title/Abstract] OR "maternal hyperglycemia*"[Title/Abstract] OR "pregnancy hyperglycemia*"[Title/Abstract] OR "caesarian section*"[Title/Abstract] OR "caesarian deliver*"[Title/Abstract] OR "cesarian deliver*"[Title/Abstract] OR "cesarian section*"[Title/Abstract] OR "mode of delivery"[Title/Abstract] OR "birth outcome*"[Title/Abstract] OR "operative deliver*"[Title/Abstract] OR "c-section*"[Title/Abstract] OR "c section*"[Title/Abstract] OR "maternal sepsis"[Title/Abstract] OR "maternal intensive care unit admission*"[Title/Abstract] OR "maternal admission* to an intensive care unit"[Title/Abstract] OR "maternal health" [Title/Abstract] OR "maternal care" [Title/Abstract] OR "neonatal care" [Title/Abstract] OR "neonatal health" [Title/Abstract] OR "newborn health" [Title/Abstract] OR "newborn care" [Title/Abstract] OR "antenatal care" [Title/Abstract] OR "prenatal care" [Title/Abstract] OR "prenatal health" [Title/Abstract] OR "postpartum health" [Title/Abstract] OR "perinatal health" [Title/Abstract] OR "perinatal care" [Title/Abstract] OR "reproductive health" [Title/Abstract] OR "premature birth*"[Title/Abstract] OR "preterm birth*"[Title/Abstract] OR "preterm deliver*"[Title/Abstract] OR "pre term birth*"[Title/Abstract] OR "birth weight" [Title/Abstract] OR "weight at birth" [Title/Abstract] OR "lbw" [Title/Abstract] OR "small for gestational age" [Title/Abstract] OR SGA[Title/Abstract] OR LGA[Title/Abstract] OR "large for gestational age" [Title/Abstract] OR "macrosomia*"[Title/Abstract] OR "stillbirth*"[Title/Abstract] OR "neonatal mortality" [Title/Abstract] OR "neonatal death*"[Title/Abstract] OR "perinatal mortality" [Title/Abstract] OR "birth malformation*"[Title/Abstract] OR "congenital malformation*"[Title/Abstract] OR "birth defect*"[Title/Abstract] OR apgar[Title/Abstract] OR "neonatal resuscitation"[Title/Abstract] OR "obstetric*"[Title/Abstract] OR "labo*r induction" [Title/Abstract] OR "induction of labo*r"[Title/Abstract] OR "induc* labo*r"[Title/Abstract] OR "antenatal immuni*ation"[Title/Abstract] OR "prenatal immuni*ation"[Title/Abstract] OR "pregnancy immuni*ation"[Title/Abstract] OR "vaccin* during pregnancy"[Title/Abstract] OR "maternal immuni*ation"[Title/Abstract] OR "antenatal vaccin*"[Title/Abstract] OR "maternal vaccin*"[Title/Abstract] OR "pregnancy screening"[Title/Abstract] OR "maternal screening"[Title/Abstract] OR "prenatal screening" [Title/Abstract] OR "prenatal testing"[Title/Abstract] OR "antenatal screening"[Title/Abstract] OR "antenatal testing"[Title/Abstract] OR "neonatal intensive care"[Title/Abstract] OR "neonatal ICU"[Title/Abstract] OR NICU[Title/Abstract] OR "newborn intensive care"[Title/Abstract] OR "epidural analgesia"[Title/Abstract] OR "epidural anesthesia"[Title/Abstract] OR "labo*r pain management"[Title/Abstract] OR "pain management during labo*r"[Title/Abstract] OR "analgesia during labo*r"[Title/Abstract] OR "pain control in labo*r"[Title/Abstract] OR "pain relief during labo*r"[Title/Abstract]) Filters: from 2000 - 2025 |
| Scopus | (Europe OR "European Union" OR EU OR "European Free Trade Association" OR EFTA OR Euro* OR Austria OR Belgium OR Bulgaria OR Croatia OR Cyprus OR "Czech Republic" OR Denmark OR Estonia OR Finland OR France OR Germany OR Greece OR Hungary OR Iceland OR Ireland OR Italy OR Latvia OR Liechtenstein OR Lithuania OR Luxembourg OR Malta OR Netherlands OR Norway OR Poland OR Portugal OR Romania OR Slovakia OR Slovenia OR Spain OR Sweden OR Switzerland OR "United Kingdom" OR UK OR England OR Scotland OR Wales OR "Northern Ireland" ) AND TITLE-ABS(((second-generation OR "second generation" OR descendant* OR children OR offspring ) AND (migrant* OR immigrant* OR "foreign-born parent*" OR refugee* )) OR "parental migration" OR "migrat* background*" OR "migrant background*" OR "family migrat* history" OR "immigrant descen*" OR "migrant descen*" ) AND (TITLE-ABS("pregnancy outcome*") OR TITLE-ABS("neonatal outcome*") OR TITLE-ABS("perinatal outcome*") OR TITLE-ABS("maternal outcome*") OR TITLE-ABS("maternal mortalit*") OR INDEXTERMS("maternal mortality") OR TITLE-ABS("maternal death*") OR INDEXTERMS("maternal death") OR TITLE-ABS("pregnancy-related mortalit*") OR TITLE-ABS("pregnancy-related death*") OR TITLE-ABS("pregnancy-associated mortalit*") OR TITLE-ABS("maternal near-miss") OR TITLE-ABS("severe maternal morbidit*") OR TITLE-ABS("severe acute maternal morbidit*") OR TITLE-ABS(eclampsia*) OR INDEXTERMS(eclampsia) OR TITLE-ABS(preeclampsia*) OR TITLE-ABS("pre eclampsia*") OR TITLE-ABS("Pregnancy toxemia*") OR TITLE-ABS("toxemia of pregnanc*") OR INDEXTERMS(pre-eclampsia) OR TITLE-ABS("gestational diabete*") OR TITLE-ABS("maternal diabete*") OR TITLE-ABS("pregnancy diabete*") OR TITLE-ABS("gestational hyperglycemia") OR TITLE-ABS("maternal hyperglycemia*") OR TITLE-ABS("pregnancy hyperglycemia*") OR TITLE-ABS("caesarian section*") OR TITLE-ABS("caesarian deliver*") OR TITLE-ABS("cesarian deliver*") OR TITLE-ABS("cesarian section*") OR TITLE-ABS("mode of delivery") OR TITLE-ABS("birth outcome*") OR TITLE-ABS("operative deliver*") OR TITLE-ABS(c-section*) OR TITLE-ABS("c section*") OR TITLE-ABS("maternal sepsis") OR TITLE-ABS("maternal intensive care unit admission*") OR TITLE-ABS("maternal admission* to an intensive care unit") OR TITLE-ABS("maternal health") OR TITLE-ABS("maternal care") OR TITLE-ABS("neonatal care") OR TITLE-ABS("neonatal health") OR TITLE-ABS("newborn health") OR TITLE-ABS("newborn care") OR TITLE-ABS("antenatal care") OR TITLE-ABS("prenatal care") OR TITLE-ABS("prenatal health") OR TITLE-ABS("postpartum health") OR TITLE-ABS("perinatal health") OR TITLE-ABS("perinatal care") OR TITLE-ABS("reproductive health") OR TITLE-ABS("premature birth*") OR TITLE-ABS("preterm birth*") OR TITLE-ABS("preterm deliver*") OR TITLE-ABS("pre term birth*") OR TITLE-ABS("birth weight") OR TITLE-ABS("weight at birth") OR TITLE-ABS(lbw) OR TITLE-ABS("small for gestational age") OR TITLE-ABS(SGA) OR TITLE-ABS(LGA) OR TITLE-ABS("large for gestational age") OR TITLE-ABS(macrosomia*) OR TITLE-ABS(stillbirth*) OR TITLE-ABS("neonatal mortality") OR TITLE-ABS("neonatal death*") OR TITLE-ABS("perinatal mortality") OR TITLE-ABS("birth malformation*") OR TITLE-ABS("congenital malformation*") OR TITLE-ABS("birth defect*") OR TITLE-ABS(apgar) OR TITLE-ABS("neonatal resuscitation") OR TITLE-ABS(obstetric*) OR TITLE-ABS("labo*r induction") OR TITLE-ABS("induction of labo*r") OR TITLE-ABS("induc* labo*r") OR TITLE-ABS("antenatal immuni*ation") OR TITLE-ABS("prenatal immuni*ation") OR TITLE-ABS("pregnancy immuni*ation") OR TITLE-ABS("vaccin* during pregnancy") OR TITLE-ABS("maternal immuni*ation") OR TITLE-ABS("antenatal vaccin*") OR TITLE-ABS("maternal vaccin*") OR TITLE-ABS("pregnancy screening") OR TITLE-ABS("maternal screening") OR TITLE-ABS("prenatal screening") OR TITLE-ABS("prenatal testing") OR TITLE-ABS("antenatal screening") OR TITLE-ABS("antenatal testing") OR TITLE-ABS("neonatal intensive care") OR TITLE-ABS("neonatal ICU") OR TITLE-ABS(NICU) OR TITLE-ABS("newborn intensive care") OR TITLE-ABS("epidural analgesia") OR TITLE-ABS("epidural anesthesia") OR TITLE-ABS("labo*r pain management") OR TITLE-ABS("pain management during labo*r") OR TITLE-ABS("analgesia during labo*r") OR TITLE-ABS("pain control in labo*r") OR TITLE-ABS("pain relief during labo*r")) AND PUBYEAR > 1999 |
| Embase | ((Europe or "European Union" or EU or "European Free Trade Association" or EFTA or Euro* or Austria or Belgium or Bulgaria or Croatia or Cyprus or "Czech Republic" or Denmark or Estonia or Finland or France or Germany or Greece or Hungary or Iceland or Ireland or Italy or Latvia or Liechtenstein or Lithuania or Luxembourg or Malta or Netherlands or Norway or Poland or Portugal or Romania or Slovakia or Slovenia or Spain or Sweden or Switzerland or "United Kingdom" or UK or England or Scotland or Wales or "Northern Ireland") and (((second-generation or "second generation" or descendant* or children or offspring) and (migrant* or immigrant* or "foreign-born parent*" or refugee*)) or "parental migration" or "migrat* background*" or "migrant background*" or "family migrat* history" or "immigrant descen*" or "migrant descen*")).mp. and (("pregnancy outcome*" or "neonatal outcome*" or "perinatal outcome*" or "maternal outcome*" or "maternal mortalit*").tw. or exp "maternal mortality"/ or "maternal death*".tw. or exp "maternal death"/ or "pregnancy-related mortalit*".tw. or "pregnancy-related death*".tw. or "pregnancy-associated mortalit*".tw. or "maternal near-miss".tw. or "severe maternal morbidit*".tw. or "severe acute maternal morbidit*".tw. or eclampsia*.tw. or exp eclampsia/ or preeclampsia*.tw. or "pre eclampsia*".tw. or "Pregnancy toxemia*".tw. or "toxemia of pregnanc*".tw. or exp pre-eclampsia/ or "gestational diabete*".tw. or "maternal diabete*".tw. or "pregnancy diabete*".tw. or "gestational hyperglycemia".tw. or "maternal hyperglycemia*".tw. or "pregnancy hyperglycemia*".tw. or "caesarian section*".tw. or "caesarian deliver*".tw. or "cesarian deliver*".tw. or "cesarian section*".tw. or "mode of delivery".tw. or "birth outcome*".tw. or "operative deliver*".tw. or c-section*.tw. or "c section*".tw. or "maternal sepsis".tw. or "maternal intensive care unit admission*".tw. or "maternal admission* to an intensive care unit".tw. or "maternal health".tw. or "maternal care".tw. or "neonatal care".tw. or "neonatal health".tw. or "newborn health".tw. or "newborn care".tw. or "antenatal care".tw. or "prenatal care".tw. or "prenatal health".tw. or "postpartum health".tw. or "perinatal health".tw. or "perinatal care".tw. or "reproductive health".tw. or "premature birth*".tw. or "preterm birth*".tw. or "preterm deliver*".tw. or "pre term birth*".tw. or "birth weight".tw. or "weight at birth".tw. or lbw.tw. or "small for gestational age".tw. or SGA.tw. or LGA.tw. or "large for gestational age".tw. or macrosomia*.tw. or stillbirth*.tw. or "neonatal mortality".tw. or "neonatal death*".tw. or "perinatal mortality".tw. or "birth malformation*".tw. or "congenital malformation*".tw. or "birth defect*".tw. or apgar.tw. or "neonatal resuscitation".tw. or obstetric*.tw. or "labo*r induction".tw. or "induction of labo*r".tw. or "induc* labo*r".tw. or "antenatal immuni*ation".tw. or "prenatal immuni*ation".tw. or "pregnancy immuni*ation".tw. or "vaccin* during pregnancy".tw. or "maternal immuni*ation".tw. or "antenatal vaccin*".tw. or "maternal vaccin*".tw. or "pregnancy screening".tw. or "maternal screening".tw. or "prenatal screening".tw. or "prenatal testing".tw. or "antenatal screening".tw. or "antenatal testing".tw. or "neonatal intensive care".tw. or "neonatal ICU".tw. or NICU.tw. or "newborn intensive care".tw. or "epidural analgesia".tw. or "epidural anesthesia".tw. or "labo*r pain management".tw. or "pain management during labo*r".tw. or "analgesia during labo*r".tw. or "pain control in labo*r".tw. or "pain relief during labo*r".tw.)  Limit to 2000-Current |

**Table S4.** Reported outcomes by study (proportion in second-generation mothers vs natives). When more than one paper from the same data source reported on the same outcome, we extracted the data from the paper with the highest number of included population.

| **Study** | **Elective c-section** | **Emergency c-section** | **Preterm birth** | **Late ANC** | **Low number of ANC visits** | **Gestational diabetes** | **Low birth weight** | **Birth weight*** | **Analgesia during labour** | **Induction of labour** | **Access to neonatal care unit** | **Maternal hypertension** | **Apgar score** | **Others** |
| --- | --- | --- | --- | --- | --- | --- | --- | --- | --- | --- | --- | --- | --- | --- |
| **Choté 2014** |  |  |  | 18.7% vs NA |  |  |  |  |  |  |  |  |  |  |
| **Sørbye 2014** |  |  |  |  |  |  |  |  |  |  |  |  |  | Stillbirth: 0.5% vs 0.3% |
| **David 2014** | Same population of David 2015 | |  |  | Same population of David 2019 |  |  |  |  |  |  |  | Same population of David 2017 |  |
| **David 2015** | 11.2% vs 15.2% | 18.8% vs 24.0% |  |  |  |  |  |  |  |  | 13.9% vs 18.9% |  |  |  |
| **Boerleider 2015** |  |  |  | 14.1% vs 6.5% | 2.0% vs 1.3% |  |  |  |  |  |  |  |  |  |
| **Reiss 2016** | Same population of David 2015 | |  |  |  |  |  |  |  | 30.9% vs 29.9% |  |  |  |  |
| **Razum 2017** | Same population of David 2015 | |  |  |  |  |  |  | 50.9% vs 63.0% |  |  |  |  |  |
| **Bakken 2017** | 1.3% vs 5.8% | 7.9% vs 8.9% | 11.8% vs 2.5% |  |  | 11.8% vs 1.1% | 6.6% vs 1.4% | 3200g vs 3600 | 47.4% vs 39.1% | 14.5% vs 12.6% | 2.6% vs 4.5% |  |  |  |
| **David 2017** | Same population of David 2015 | | Same population of David 2018 |  |  |  | 5.9% vs 8.7% | 3340g vs 3330 |  |  | Same population of David 2015 |  | 1.6% vs 2.3% | SGA: 7.9% vs 8.4% |
| **El-Khoury Lesueur 2018** |  |  |  |  |  | 7.7% vs 6.8% |  |  |  |  |  |  |  |  |
| **David 2018** | Same population of David 2015 | | 8.3% vs 10.5% |  |  |  |  |  |  |  | Same population of David 2015 |  |  |  |
| **David 2019** |  |  |  |  | 26.4% vs 23.1% | 4.3% vs 4.4% |  |  |  |  |  |  |  | Near miss: 2.2% vs 1.9% |
| **Breckenkamp 2019** | Same population of David 2015 | |  |  |  | Same population of David 2019 |  |  |  |  |  |  |  | Fetal macrosomia: 9.0% vs 11.9% |
| **Seidel 2020** |  |  |  | 18.0% vs 12.2% | 38% vs 31% |  |  |  |  |  |  |  |  |  |
| **Miani 2020** |  | 16.3% vs 22.2% |  |  |  |  |  |  |  |  |  |  |  |  |
| **Aradhya 2022** |  |  |  |  |  |  | 3.2% vs 3.3% | 3463g vs 3494 |  |  |  |  |  |  |
| **Wändell 2023** |  |  |  |  |  | Same population of Wändell 2024 |  |  |  |  |  | Same population of Wändell 2024 |  |  |
| **Lee 2023** | 8.8% vs 12.6% | 21.3% vs 20.7% | 13.8% vs 9.0% |  | 23.7% vs 20.0% |  |  | 3327g vs 3410 | 28.1% vs 20.0% | 15.6% vs 16.3% | 19.4% vs 11.9% |  | 0.0% vs 1.3% |  |
| **Wändell 2024** |  |  |  |  |  | 2.2% vs 1.7% |  |  |  |  |  | 6.0% vs 7.0% |  |  |

*Mean/median birth weight in grams

**Table S5.** Risk of bias assessment for cross-sectional studies

| **Author and year** | **Criteria for inclusion specified** | **Subjects and setting described** | **Exposure measured in a valid way** | **Objective criteria for condition** | **Confounding factors identified** | **Strategies to deal with confounding factors stated** | **Outcomes measured in a valid way** | **Appropriate statistical analysis** | **Overall appraisal and notes** |
| --- | --- | --- | --- | --- | --- | --- | --- | --- | --- |
| Choté 2014 | y | y | y | NA | y | y | y | y | Differences in % missing in independent variables between groups. Possible selection bias due to lack of informed consent |
| David 2014 | y | y | y | NA | y | y | y | y |  |
| David 2015 | y | y | y | NA | y | y | y | y |  |
| Reiss 2016 | y | y | y | NA | y | y | y | y |  |
| Razum 2017 | y | y | y | NA | y | y | y | y |  |
| David 2017 | y | y | y | NA | y | y | y | y |  |
| David 2018 | y | y | y | NA | y | y | y | y |  |
| David 2019 | y | y | y | NA | y | y | y | y |  |
| Breckenkamp 2019 | y | y | y | NA | y | y | y | y |  |
| Seidel 2020 | y | y | y | NA | y | y | y | y | Low response rate (58%) |
| Lee 2023 | y | y | y | NA | y | y | y | y | Possible selection bias due to language |

**Table S6.** Risk of bias assessment for cohort studies

| **Author and publication year** | **Groups recruited from the same population** | **Exposures measured similarly for exposed and unexposed** | **Exposure measured in a valid way** | **Confounding factors identified** | **Strategies to deal with confounding factors stated** | **Participants free of the outcome at the start or at exposure** | **Outcomes measured in a valid way** | **Follow up time reported and sufficient for outcomes** | **Follow up complete (or reasons to loss)** | **Strategies to address incomplete follow up** | **Appropriate statistical analysis** | **Overall appraisal and notes** |
| --- | --- | --- | --- | --- | --- | --- | --- | --- | --- | --- | --- | --- |
| Sørbye 2014 | y | y | y | y | y | y | y | y | y | y | y |  |
| Boerleider 2015 | y | y | y | y | y | y | y | y | y | N | y | Low response rate and low sample of SGMs |
| Bakken 2017 | y | y | y | y | y | y | y | NA | NA | NA | y/n | Low power due to small sample size |
| El-Khoury Lesueur 2018 | y | y/n | y | y | y | NA | y | y | y | n | y | Migration status missing for 7% of population |
| Miani 2020 | y | y | y | y | y | y | y | NA | y | n | y | Possible selection bias (due to languages used in the questionnaire and recruitment method) |
| Aradhya 2022 | y | y | y | y | y | y | y | y | NA | NA | y |  |
| Wändell 2023 | y | y | y | y | y | y | y | y | y | NA | y |  |
| Wändell 2024 | y | y | y | y | y | y | y | y | y | n | y |  |
